# Spanish-Language Patient Education Materials for Obstetric Anesthesia: A Comparison of Readability and Quality of Online Spanish-Language Resources

**DOI:** 10.1177/26884844251394823

**Published:** 2025-11-10

**Authors:** Mariana Restrepo, Sananda Pai, Talia Scott, Garrett W. Burnett

**Affiliations:** Department of Anesthesiology, Perioperative and Pain Medicine, Icahn School of Medicine at Mount Sinai, New York, New York, USA.

**Keywords:** obstetric anesthesia, patient education materials, quality analysis, readability analysis

## Abstract

**Introduction:**

Thousands of Hispanic parturients give birth in the United States annually, necessitating accessible health education resources in Spanish. Given the known Hispanic maternal care disparities and high reading levels of Spanish-written patient education materials (PEMs), this study aims to assess the readability and quality of obstetric (OB) anesthesia Spanish-language PEMs from a general internet search and academic leaders. We hypothesize that the readability and quality of PEMs from academic leaders will be superior to those found *via* general internet search.

**Methods:**

To identify Spanish-written PEMs on OB anesthesia, the webpages of 62 academic medical centers (AMCs) recognized as OB anesthesia leaders were screened. A general internet search using “anestesia y alivio del dolor durante el parto” (“anesthesia and pain relief during labor and delivery”) was conducted to find an equal number of additional resources. Readability was assessed using the Fernandez-Huerta Readability Index (FHRI) and Indice de Legibilidad de Flesch-Szigriszt (INFLESZ) analyses, while quality was evaluated using the DISCERN instrument and the Health Education Materials Assessment Tool (HEMAT).

**Results:**

Twenty-eight Spanish-language PEMs from AMCs and 28 from a general internet search were identified. The FHRI and INFLESZ readability analyses revealed that PEMs from both cohorts primarily aligned with a 9–10th grade reading level. These reading levels significantly exceeded the recommended 4–6th grade level (*p* < 0.001). DISCERN scores indicated no quality difference between cohorts. Both groups achieved high HEMAT scores for understandability.

**Conclusion:**

The readability of online OB anesthesia Spanish-written PEMs from AMCs and a general internet search was both similar and higher than recommended. Quality did not differ between both cohorts. Improvements in readability and quality are needed for better patient-centered care and to emphasize the importance of shared decision-making.

## Introduction 

Approximately 25%, or 890,000, of all live births in the United States are Hispanic parturients.^1^ Therefore, it is imperative that online Spanish-language patient education materials (PEMs) regarding obstetric anesthesia be informative and readable by Spanish-speaking patients.

Readability, or “the objective measurement of the reading skills one should possess to understand the written material,” is quantifiable by several measures.^2^ The American Medical Association and the Agency for Healthcare Research and Quality currently recommend PEMs be written at a 4–6th grade reading level for optimal patient comprehension.^3,4^ Exceeding this may limit patients’ understanding of important health information due to potential literacy barriers. Most English-written PEMs, including those for obstetric anesthesia, are known to be written at an 11th grade or higher reading level.^5,6^

Little is known about the readability of online Spanish-language PEMs for obstetric anesthesia available *via* online search, but previous literature demonstrates that 82.5% of internet-based Spanish-language PEMs for various disciplines exceed current recommended reading levels.^7^ Similarly, academic medical centers (AMCs) in the United States have been found to have Spanish-language PEMs on labor analgesia written at an 8–11th grade reading level and lack adequate information on risks and benefits.^8^ Such shortcomings may have important implications, as a lack of education on labor analgesia correlates with lower odds of receiving labor analgesia for Hispanic parturients.^9,10^ Vaginal delivery patients who did not receive epidural analgesia either chose parenteral analgesia or forwent pain relief altogether. Increasing the readability and quality of online Spanish-language PEMs on obstetric anesthesia is therefore vital to improve equitable knowledge and care.

Hispanic patients are more likely to rely on online resources for health information; therefore, internet-based Spanish-language PEMs must provide readable and high-quality content for all patients.^6^ Currently, the readability and quality of online search results for Spanish-language PEMs on obstetric anesthesia are unknown. This study therefore aims to analyze the readability and quality of Spanish-language PEMs on obstetric anesthesia by comparing those written by leading AMCs in obstetric anesthesia with those readily accessed by a general internet search. We hypothesize that the readability and quality of PEMs from AMCs will be superior to those found *via* general internet search.

## Methods

### Search methods

Spanish-language obstetric anesthesia PEMs published on the websites of institutions designated as centers of excellence (COE) by the Society for Obstetric Anesthesia and Perinatology were accessed.^11^ We chose these AMCs as the model for patient education, considering COE are leaders in obstetric anesthesia. A second group of readily accessible internet-sourced PEMs on labor analgesia was compiled *via* an internet search using Google (Alphabet Inc., Mountain View, CA). This search engine was chosen as it holds approximately 90% of the search engine market share, and therefore represents the most commonly used search engine.^12^ Thus, we compared two categories of online Spanish-language PEMs, AMC PEMs and general internet search PEMs.

To identify Spanish-language AMC PEMs, two authors fluent in English and Spanish (M.R. and S.P.) screened the websites for all COE (*n* = 62) to identify relevant online PEMs.^13^ PEMs were included if they were accessed *via* links to Spanish-language materials available on the COE websites or through a link to the Spanish-language version of the COE website. COE PEMs that were automatically translated from English by the internet browser were excluded, as were videos or infographic flyers without information written in complete sentences. This screening of COE websites resulted in a total of 28 Spanish-language AMC PEMs.

To identify Spanish-language general internet search PEMs, the same authors (M.R. and S.P.) performed an internet search using Google on September 9, 2024, using one search term “anestesia y alivio del dolor durante el parto” (in English, “anesthesia and pain relief during labor and delivery”). This search term represents a common inquiry of patients prior to labor and delivery. A private browsing mode using a clean browser that was not connected to any accounts and lacked any browser history was used to ensure an unbiased search. Websites were excluded if they pertained to a medical institution outside of the United States, were published in a scientific journal, were written for medical professionals, or only contained videos. Nine search results from both the AMC search and the general internet search were duplicates, therefore representing the same PEMs. These were retained within the comparison groups to reflect the primary resources available to patients on the internet. Search results were screened consecutively until 28 PEMs were identified, resulting in an equal number of PEMs to allow for a fair comparison between AMCs and a general internet search.

### Readability analysis (primary outcome)

Readability of each article was assessed by two different tools, the Fernandez-Huerta Readability Index (FHRI) and the Indice de Legibilidad de Flesch-Szigriszt (INFLESZ), both of which have been validated for assessing the readability of PEMs. These scores were calculated using an online readability calculator for each PEM.^14^ The FHRI, a Spanish adaptation of the Flesch Reading-Ease score for English texts, uses total word, sentence, and syllable counts to create a score on a 0–100 scale, with higher scores indicating easier readability.^15^ The INFLESZ score similarly takes into account the total word, sentence, and syllable counts to determine readability using the following scale: “Very difficult” (<80), “Somewhat difficult” (40–55), “Normal” (55–65), “Quite easy” (65–80), and “Very easy” (>80).^16,17^

### Quality analysis (secondary outcome)

The quality of each PEM with respect to informational content and overall understandability was assessed using two validated and reliable metrics that measure the quality of written health information, the DISCERN instrument and the Health Education Materials Assessment Tool (HEMAT).

The DISCERN instrument is a survey that incorporates 15 questions on the reliability and one question on the overall quality of a PEM.^18^ The first eight questions reflect the extent to which the information can be trusted and is reliable, while the next seven refer to the informational content of the PEM. The final question asks the user of the instrument to consider the previous 15 questions to determine the overall quality of the PEM. All questions are answered on a 1–5 scale, with 1 representing a complete lack of criteria being fulfilled, 3 representing partial completion of criteria, and 5 representing full completion of criteria. Scores 2 and 4 are assigned if the PEM falls between scores 1 and 3, and 3 and 5, respectively.

The HEMAT is an adaptation of the Agency for Healthcare Research and Quality’s Patient Education Materials Assessment Tool that specifically focuses on the understandability of PEM content and has been validated for Spanish-language materials.^19^ This analysis consists of six yes-or-no questions on the purpose, wording, and organization of the text. The HEMAT score is representative of how many criteria were met out of the total relevant criteria.

For the quality analysis, two authors (M.R. and S.P.) separately assigned DISCERN and HEMAT scores for each PEM by answering each quality analysis question. The scores assigned to each individual question were then averaged across the two raters to obtain final quality scores that were used in the statistical analysis.

### Statistical analysis

Readability scores were calculated as raw continuous scores or as grade levels. The FHRI and INFLESZ analyses yielded continuous readability scores, which were converted to categorical grade levels using the respective conversion tables (Supplementary Table S1). These were also summarized using proportions and percentages.

The independent *t*-test was used to compare the average readability scores of PEMs from AMCs to those from the general online search. Furthermore, the average FHRI and INFLESZ scores for all PEMs were compared with the nationally recommended reading level using the one-sample *t*-test. The authors used a 5th grade reading level for this statistical comparison, since the AMA recommends a reading level range from the 4th to 6th grade.^3,20^ The average FHRI and INFLESZ scores were specifically compared with scores of 85 and 72, which are respectively representative of the 5th grade level (Supplementary Table S1). Fisher’s exact test was used to analyze the proportions of grade levels of both groups.

The mean and standard deviation of the final DISCERN and HEMAT scores were computed, both for the individual questions and the total scores of each analysis. The total DISCERN score was represented as a percentage of the maximum score of 80 points. Currently, there is no gold standard for the reporting and analysis of DISCERN scores; therefore, the authors used means and standard deviations, similar to previous studies.^21^ The HEMAT scores as percentages are reflective of a total of 6 possible points. All continuous DISCERN and HEMAT scores were compared between the AMC and general internet search cohorts using the independent *t*-test, with *p* < 0.05 indicating statistical significance. To assess the inter-rater reliability of the scores assigned by both authors, Cohen’s kappa was determined for the DISCERN and HEMAT analyses. Statistical analyses were completed using SPSS Statistics (v29.0.2; Armonk, NY).

## Results

### Search results

**FIG. 1.**
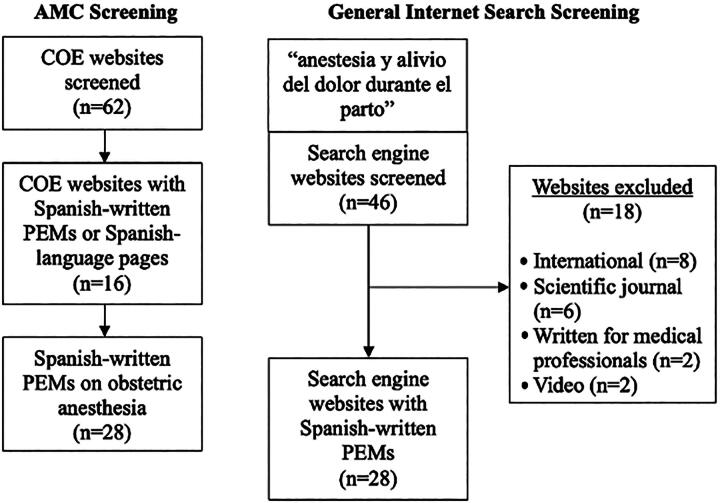
Screening process for academic medical centers (AMCs) and general internet search PEMs.

A total of 62 AMCs were screened for Spanish-written online resources for obstetric anesthesia patients. Twenty-eight online articles originally written for Spanish-speaking audiences were collected from 16 AMCs. In regard to PEMs from a general internet search, 28 out of 46 total materials screened for inclusion were selected by two authors (M.R. and S.P.) (Fig. 1). Websites were excluded (*n* = 18) if they pertained to a medical institution outside of the United States (*n* = 8), were published in a scientific journal (*n* = 6), were written for medical professionals (*n* = 2), or contained solely videos (*n* = 2). Nine duplicate search results between the AMCs and the general internet search were retained to reflect the primary resources available to patients on the internet.

### Readability analysis, Fernandez-Huerta grade level

On average, materials from AMCs had an FHRI of 57.7, which indicates a 9–10th grade reading level (Table 1). PEMs from the general internet search resulted in an average FHRI of 58.9, which also indicates a 9–10th grade reading level (Table 1). There was no statistically significant difference between the average readability indices of these two cohorts (*p* = 0.476). When comparing the average FHRI scores of AMCs and the general internet search to a 4–6th grade reading level, they were both significantly higher (*p* < 0.001).

**Table 1.** Readability Analysis Using Fernandez-Huerta and Indice de Legibilidad de Flesch-Szigriszt Grade Levels and Mean Scores for Patient Education Materials from Academic Medical Centers and a General Internet Search

	Mean (SD)	Grade level
AMC		
Fernandez-Huerta grade level	57.7 (7.5)	9–10th grade
INFLESZ grade level	53.0 (7.5)	9–10th grade
General internet search		
Fernandez-Huerta grade level	58.9 (5.4)	9–10th grade
INFLESZ grade level	54.3 (5.5)	9–10th grade

Both analyses use total word, sentence, and syllable counts to calculate a readability score. Grade levels were extrapolated from individual mean scores using each test. All comparisons between AMCs and the general internet search were statistically insignificant (*p* > 0.05).

AMC, academic medical center; INFLESZ, Indice de Legibilidad de Flesch-Szigriszt; SD, standard deviation.

### Readability analysis, INFLESZ grade level

The INFLESZ analysis of PEMs from AMCs demonstrated an average score of 53.0, which is reflective of a “difficult” or 9–10th grade reading level (Table 1). When analyzing PEMs from a general internet search, the average INFLESZ score was 54.3, which is similarly indicative of a “difficult” or 9–10th grade reading level (Table 1). There were no significant differences between the PEMs from AMCs and the general internet search (*p* = 0.476). Both the average scores for AMCs (*p* < 0.001) and the general internet search (*p* < 0.001) were significantly higher than the recommended 4–6th grade reading level.

### DISCERN

Overall, there was no statistically significant difference between the total DISCERN scores of the AMC and general internet search PEMs (*p* = 0.077; Table 2). The average DISCERN score for the AMC PEMs resulted in a 3.38, while that for the general internet search PEMs resulted in a 3.69. Both indicate partial completion of quality criteria (*p* = 0.077; Table 2). When analyzing the individual DISCERN questions, general internet search PEMs scored significantly higher on those related to the clarity of aims (*p* < 0.001), information source (*p* < 0.001), information date of origin (*p* = 0.001), and treatment description (*p* < 0.001) when compared with AMC PEMs (Supplementary Table S2). This reflects a greater completion of question-specific criteria for general internet search PEMs. Inter-rater reliability of the scores was κ = 0.332 (*p* < 0.001), which reflects fair agreement between authors.

**Table 2.** Quality Analyses of Patient Education Materials from Academic Medical Centers and a General Internet Search Using DISCERN and Health Education Materials Assessment Tool

	Mean (SD)		
	AMC	General internet search	*p*-Value
DISCERN			
Average score	3.38 (0.52)	3.69 (0.38)	0.077
Percentage	67.7 (10.3)	73.8 (7.5)	0.077
HEMAT			
Average score	5.29 (0.50)	5.54 (0.47)	0.572
Percentage	88.1 (8.31)	92.3 (7.83)	0.572

HEMAT, Health Education Materials Assessment Tool.

### HEMAT

The HEMAT analysis demonstrated no significant difference in understandability when comparing AMC and general internet search PEMs according to the percentage of points scored (88.1% vs. 92.3%; *p* = 0.572; Table 2). All PEMs analyzed in this study scored more than 70% of the possible points, indicating that most understandability criteria were met. In regard to the individual HEMAT questions, general internet search PEMs were slightly more likely to meet criteria relating to the following: lack of distracting information, material broken down into short sections, and material uses visual cues to highlight key points (*p* = 0.043; Supplementary Table S3). The inter-rater reliability between authors proved to be κ = 0.563 (*p* = 0.563), which is representative of substantial agreement.

## Discussion

Our study found that all included obstetric anesthesia PEMs intended for Spanish-speaking patients, whether created by an AMC or accessed *via* a general internet search, were written at higher than recommended reading levels. We hypothesized that leaders in obstetric anesthesia would create PEMs with more appropriate readability levels, but this was not supported by our findings. Both cohorts demonstrated 9–10th grade reading levels across various readability analyses, significantly exceeding the recommended 4—6th grade level. This contrasts with previous research demonstrating that English-written PEMs created by obstetric and medical societies scored more appropriate readability levels than those from an online search engine.^22^ The elevated reading difficulty in our study emphasizes a critical disparity that Spanish-speaking patients face that may hinder comprehension and engagement with educational material.

Our study uniquely explored the quality of Spanish-language PEMs on obstetric anesthesia using the DISCERN and HEMAT analyses.^8^ Overall quality for AMC and general internet search PEMs was low, and there was no significant difference between the groups. The DISCERN analysis demonstrated that general internet PEMs were slightly higher in quality when discussing aims, informational sources, information date of origin, and treatment descriptions. Similarly, these PEMs scored higher in using short sections, using visual cues, and limiting distracting information according to the HEMAT analysis. Therefore, PEMs written by institutions known for their obstetric anesthesia services are not inherently of a higher quality than those collected from a general internet search. This may suggest that Spanish-written PEMs do not effectively reflect the needs of the population they serve and may lead to language-based disparities in obstetric anesthesia care.

Altogether, the higher than recommended reading levels and inadequate quality scores of the PEMs may underlie the relationship between primary spoken language and the use of labor analgesia. Without the proper information, patients’ engagement in shared decision-making is put at risk, as well as their likelihood of experiencing adverse outcomes. Specifically, preferred spoken language of Spanish has been associated with decreased likelihood of receiving neuraxial analgesia of all types.^23^ Improving the readability and quality of Spanish PEMs can enhance education on pain control, leading to more informed decision-making for Spanish-speaking patients. This is especially important because studies have shown that interpreters are not always utilized in obstetric settings.^24,25^ A lack of appropriate patient education may also affect maternal outcomes in that obstetric patients with limited health literacy have been found to be at greater risk for cesarean section deliveries and perineal lacerations.^26^ Therefore, the distribution of PEMs in patients’ native languages may serve as a critical opportunity to ensure the understanding of labor analgesia options, potential side effects, or the overall process of receiving analgesia during labor.

Another critical finding was the lack of online resources for Spanish-speaking patients published by AMCs. Only 26% (*n* = 16/62) of AMC websites contained relevant Spanish-language PEMs. It is unclear if these findings are offset by offline Spanish-language PEMs or if AMCs truly lack appropriate PEMs. Without reputable resources available, Spanish-speaking parturients may have difficulties understanding their anesthetic options and providing informed consent. Previous studies describe Hispanic women anticipating using labor analgesia less than non-Hispanic White patients, potentially reflecting a lack of Spanish-language information available.^27^ While the extent to which early patient education affects decision-making is unclear, studies indicate that intentional health education on epidural analgesia during the third trimester improves comprehension of and satisfaction with epidural analgesia for English-speaking patients.^28^ Expanding the availability of Spanish-language PEMs could similarly impact obstetric anesthesia care for this population.

The scarcity of Spanish-language resources underscores the need for future studies to identify patients’ sources for obstetric anesthesia information. Studies suggest many Hispanic patients are unaware of anesthesiologists’ role in health care.^29^ This may discourage patients from directly approaching anesthesiologists with questions or concerns about their options and lead them to rely on other sources for information. While this study evaluated two common sources of Spanish-language PEMs, other sources of information on labor analgesia include consultations with medical professionals, social media, family, friends, and more. Additional research is needed to clarify patients’ first and primary sources for anesthetic information in order to identify areas for improvement for Spanish-language PEMs.

In terms of limitations, this study excluded images or videos from the materials and webpages collected since the readability tests solely analyze written text. Past studies highlighted how patient education, including video-based resources, results in decreased patient anxiety, increased patient satisfaction, and higher rates of opting for epidural analgesia.^30,31^ Future research should therefore investigate the adjunctive use of video with written PEMs and how it impacts general understanding of and risks associated with labor anesthesia techniques. Furthermore, the effect of images in PEMs on patient comprehension and education should be analyzed. This study also focused solely on online PEMs and therefore may not include all PEMs distributed by AMCs for obstetric anesthesia patients. PEMs may also be physically distributed to patients or shared privately online by providers. It is of note that the inter-reader reliability of the DISCERN quality analysis reflected fair agreement between the two authors, indicating some variability in subjective assessment, which may have affected the consistency of the quality scores.

In conclusion, there is a demonstrated need for improved Spanish-language obstetric anesthesia education, as the readability and content of readily available online materials may not sufficiently reflect the needs of this potentially vulnerable population. Without high-quality Spanish-language PEMs, Hispanic patients may have decreased access to analgesic options and experience more discomfort, leading to language-based disparities for this population. With more patients seeking medical information online, there is an opportunity to fill this gap by creating high-quality, readable, and accessible Spanish-language PEMs on obstetric anesthesia.

## Data Availability

The data used in this study are available to be shared upon reasonable request.

## Abbreviations Used

AMCs: Academic Medical Centers
COE: Centers of Excellence
FHRI: Fernandez-Huerta Readability Index
HEMAT: Health Education Materials Assessment Tool
INFLESZ: Indice de Legibilidad de Flesch-Szigriszt
PEMs: Patient Education Materials
