# Supplementary material for: Spanish-Language Patient Education Materials for Obstetric Anesthesia: A Comparison of Readability and Quality of Online Spanish-Language Resources
Source: Womens Health Rep (New Rochelle). 2025 Nov 10;6(1):1209–15. doi: 10.1177/26884844251394823 (PMC12726835; doi:10.1177/26884844251394823)
Supplement: Supplementary Table S1 [file 26884844251394823_supplementary_table_s1.docx]

**Supplemental Table 1.** Readability analyses and their grade-level conversions.

| **Readability Analysis** | **Grade-Level Conversion** |
| --- | --- |
| **Fernandez-Huerta Readability Index** | \| **Score** \| **Grade Level** \| \| --- \| --- \| \| 90-100 \| 4^th^ grade \| \| 80-90 \| 5^th^ grade \| \| 70-80 \| 6^th^ grade \| \| 60-70 \| 7^th^ to 8^th^ grade \| \| 50-60 \| 9^th^ to 10^th^ grade \| \| 30-50 \| 11^th^ to 12^th^ grade \| \| 0-30 \| College \| |
| Indice de Legibilidad de Flesch-Szigriszt (INFLESZ) | \| **Score** \| **Difficulty** \| \| --- \| --- \| \| <40 \| Very difficult (scientific materials) \| \| 40-55 \| Difficult (high school textbook) \| \| 55-65 \| Normal (10^th^ to 12^th^ grade textbook) \| \| 65-80 \| Easy (4^th^ to 6^th^ grade reading material) \| \| >80 \| Very easy (1^st^ to 3^rd^ grade texts) \| |
