# Supplementary figures and images for: Spanish-Language Patient Education Materials for Obstetric Anesthesia: A Comparison of Readability and Quality of Online Spanish-Language Resources
Source: Womens Health Rep (New Rochelle). 2025 Nov 10;6(1):1209–15. doi: 10.1177/26884844251394823 (PMC12726835; doi:10.1177/26884844251394823)

**Supplemental Table 2. DISCERN quality analysis.**


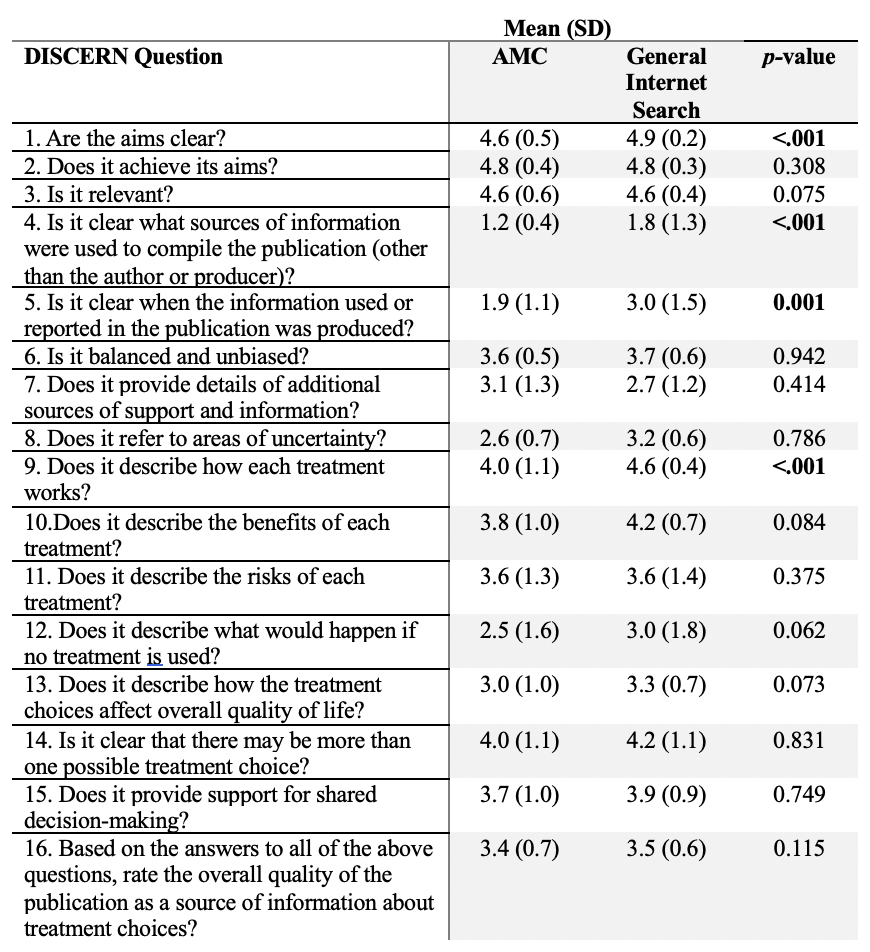

Supplement: Supplementary Table S2 [file 26884844251394823_supplementary_table_s2.docx]

**Supplemental Table 3. HEMAT quality analysis.**


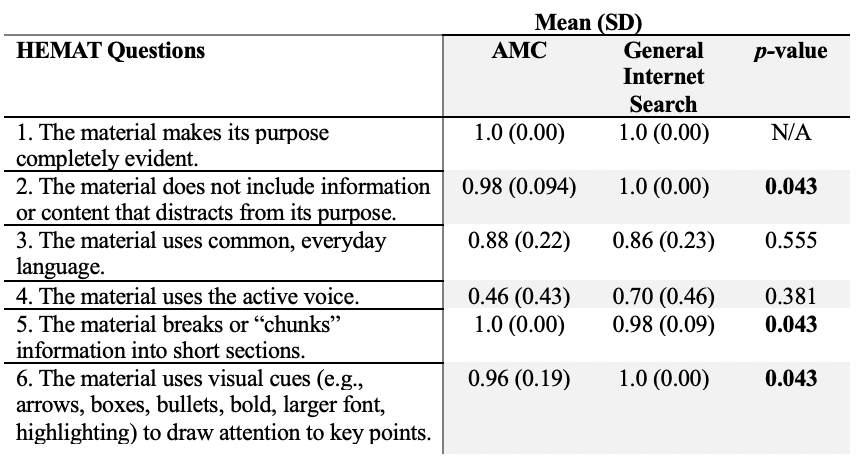

Supplement: Supplementary Table S3 [file 26884844251394823_supplementary_table_s3.docx]
